# A rare case of diploic venous anomaly: asymptomatic venous sac expanding in the diploe

**DOI:** 10.1186/s40064-016-3607-1

**Published:** 2016-11-07

**Authors:** Hirokazu Iwamuro, Shunsuke Ikeda, Makoto Taniguchi

**Affiliations:** 1 Department of Neurosurgery, Tokyo Metropolitan Neurological Hospital, 2-6-1 Musashidai, Fuchu, 183-0042 Japan; 2 Department of Research and Therapeutics for Movement Disorders, Juntendo University Graduate School of Medicine, 2-1-1 Hongo, Bunkyo-ku, Tokyo, 113-8421 Japan

**Keywords:** Intradiploic varix, Sinus pericranii, Subepicranial varix, Varix, Diploic vein, Superior sagittal sinus, Venous sac, Surgery

## Abstract

**Background:**

Vascular anomalies accompanied with the diploic veins are rare. Among them, sinus pericranii, which is characterized by abnormal connections between intra- and extracranial venous systems, is relatively common. Besides sinus pericranii, a few cases of subepicranial varix with connections to diploic veins have been reported, but these varices had no connections to intracranial venous sinuses. Herein, we present a rare case of an expanding venous sac in the diploe which communicated with the intracranial sinus but not with the extracranial venous systems.

**Case presentation:**

An adult woman presented to us with a minor transient headache. Although no abnormal appearances were found on her scalp, imaging studies showed a club-shaped venous sac in the left parietal diploe that communicated with the superior sagittal sinus and diploic veins on the medial and lateral sides, respectively. It was revealed that the lesion had expanded as compared with a previous computed tomography image. Surgery was performed to intercept venous supply from the diploic veins, and the lesion was filled with thrombi. In a follow-up of 15 months, there was no recurrence of abnormal venous flow. Histological examination showed the endothelial lining in the membranous wall of the sac, which is typically observed in sinus pericranii. However, no communication with the pericranial veins of the scalp was identified on the imaging studies and intraoperative observation. Accordingly, it was diagnosed as another entity “intradiploic varix”.

**Conclusions:**

The abnormal connection between the intracranial and the diploic venous systems via the large venous sac was surgically treated. It was pathologically similar to sinus pericranii, but anatomically considered to be another form of venous anomaly. In cases of expanding lesions, surgical treatment is recommended.

## Background

The diploic venous system is located between two compact tables of the skull. It communicates with the dural sinuses and pachymeningeal veins along the inner table, and with the pericranial veins on the outer table via the emissary veins (Hershkovitz et al. 1999). The diploic vein can be involved in various vascular anomalies although they are rare. Among these vascular anomalies, sinus pericranii is relatively common (Akram et al. 2012). Sinus pericranii typically presents as a soft swelling in the scalp which enlarges and deflates in response to increase and decrease in the intracranial pressure, respectively. It is characterized by abnormal connections between intra- and extracranial venous systems (Akram et al. 2012). Here in our report, we present a rare case of an expanding venous sac in the diploe that resembled sinus pericranii but did not communicate with the pericranial veins of the scalp. We also discuss the pathology of sinus pericranii.

## Case presentation

A 76-year-old woman with no previous medical history, including that of head trauma, presented to us with a minor transient headache and was examined. The results of the patient’s general and neurological examinations were normal. No abnormal appearances were found on her scalp.

**Fig. 1:**
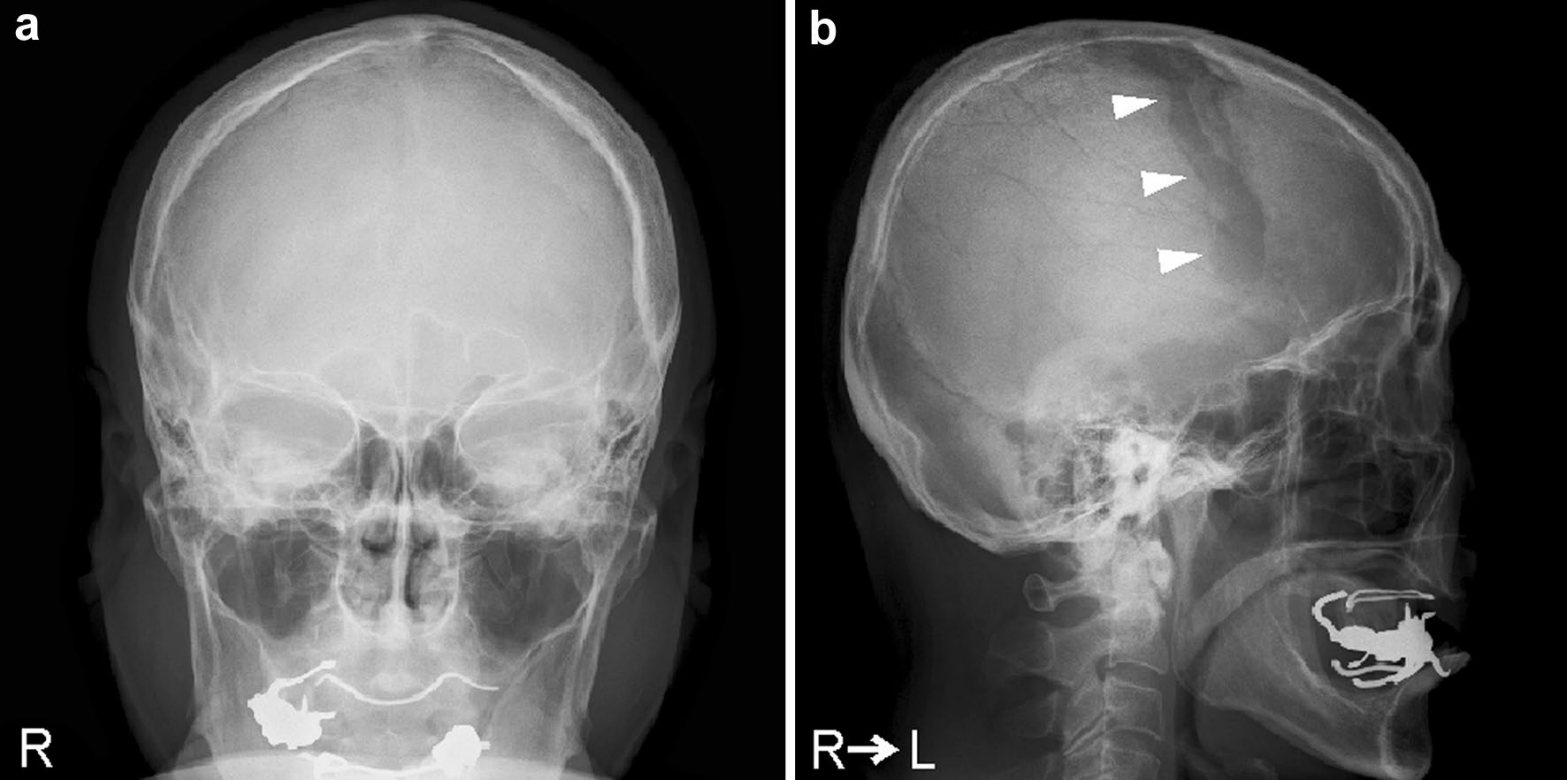
Skull radiographic images in antero-posterior view (**a**) and lateral view (**b**). A club-shaped lucent bone defect (*arrowheads*) was visible in the lateral view

**Fig. 2:**
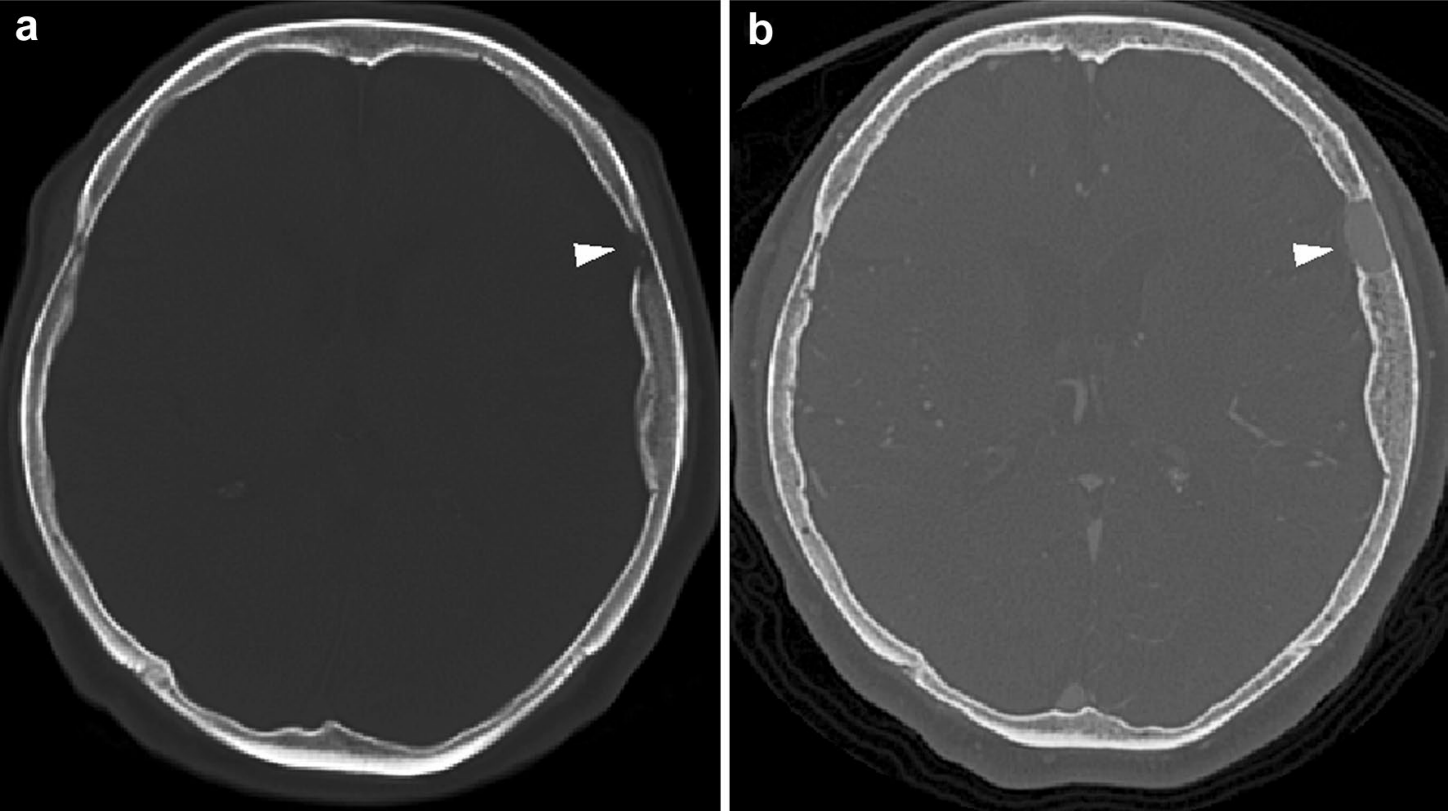
CT images 5-years prior to the operation (**a**) and immediately prior to the operation (**b**). A lesion with a bone defect in the left parietal bone expanded over 5 years (*arrowheads*)

**Fig. 3:**
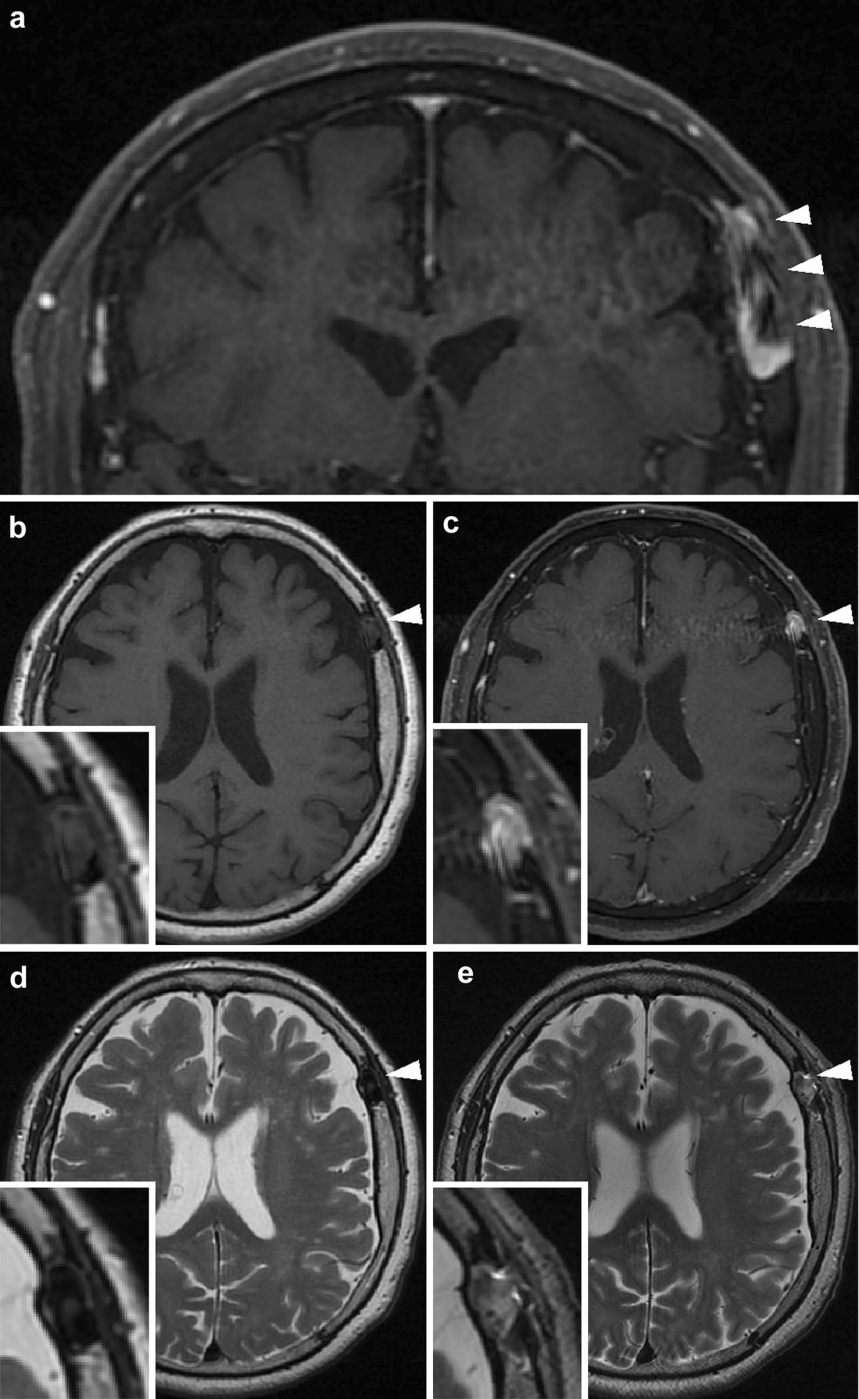
MRI scans prior to the operation (**a**–**d**) and after the operation (**e**). Each image showed a lesion (*arrowheads*) in the left parietal bone. A magnified clipping of the lesion is accompanied in the rectangle with each axial image (**d**, **e**). The inside of the lesion appeared with mixed intensity on T1- (**b**) and T2-weighted (**d**) images, and a part of the lumen was clearly enhanced with gadolinium contrast media (**a**, **c**). The intensity of the inside showed as high intensity on T2-weighted images after the operation (**e**)

**Fig. 4:**
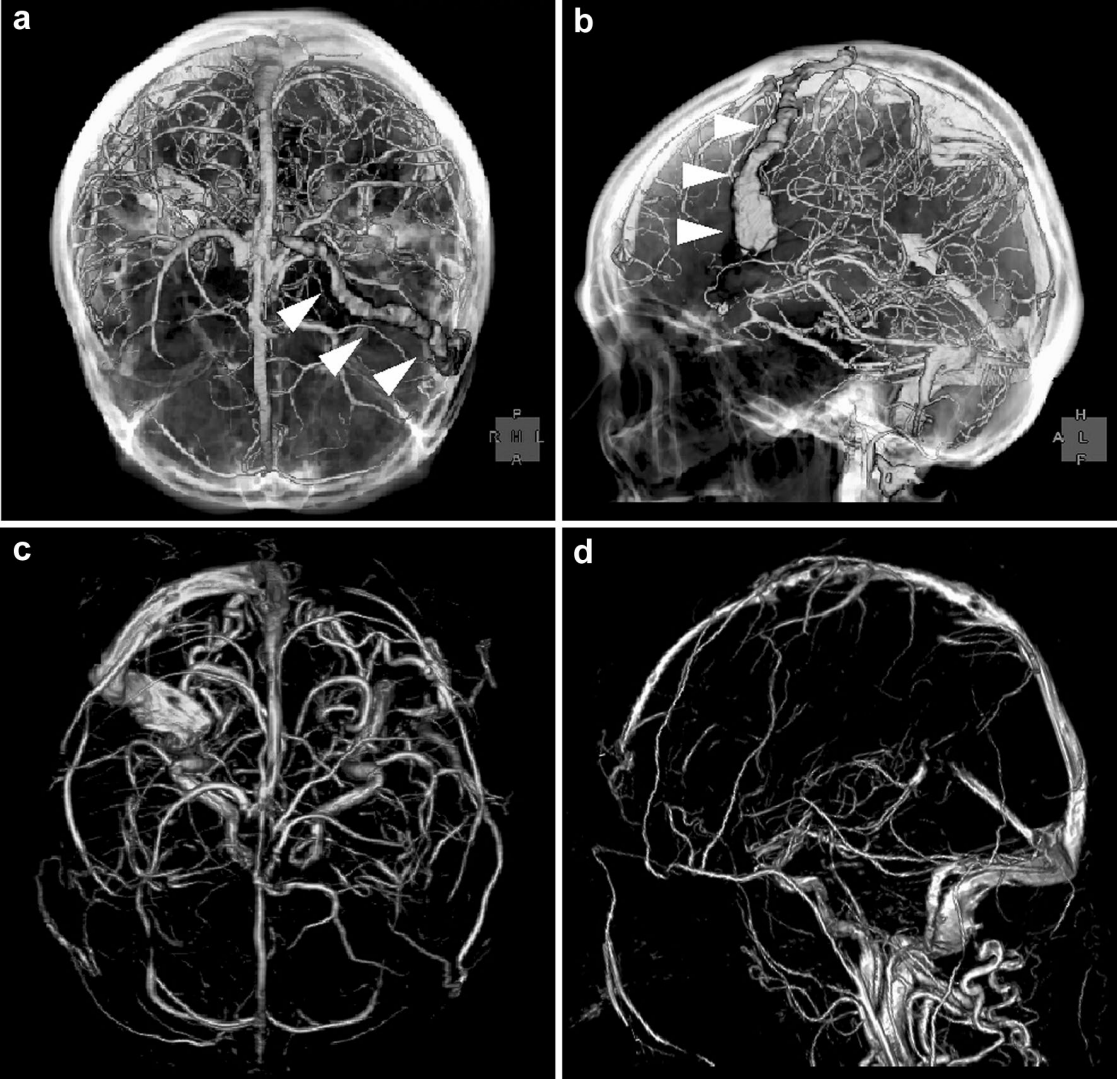
Reconstructed three-dimensional images of pre-operative CT angiography (**a**, **b**) and post-operative magnetic resonance venography (**c**, **d**). A club-shaped lumen filled with blood (*arrowheads*) disappeared after ligation of feeding diploic veins

Radiography showed the presence of a linear bone deficit in the left parietal diploe (Fig. 1). The lesion measured 1 cm × 8 cm in size, and was located almost parallel to the coronal suture. Computed tomography (CT) imaging identified hyperosteosis in the skull around the bone deficit, but the lesion did not yet eroded the outer layer of the skull (Fig. 2b). After comparing this CT image with a previous one that had been obtained 5 years before at another hospital (Fig. 2a), we determined that the lesion had expanded. Magnetic resonance imaging (MRI) of the brain was performed to examine the contents of the lesion. The lesion had an outer membrane that was iso-intensity on both T1- (Fig. 3b) and T2-weighted (Fig. 3d) images and was slightly enhanced with gadolinium contrast media (Fig. 3c). The main part of the inside appeared at iso- and low intensities on T1- (Fig. 3b) and T2-weighted (Fig. 3d) images, respectively, and it was clearly enhanced with gadolinium contrast media to the same extent as vessel lumens (Fig. 3a, c). As the results of these studies suggested the presence of a vascular abnormality, CT angiography was performed. In the 3D reconstructed images, a club-shaped lesion was visible in the venous phase (Fig. 4a, b). At the medial end, the lesion passed through the inner table of the skull and drained into the superior sagittal sinus. However, the lateral side did not appear to communicate with any vessels, and it did not cross the coronal suture.

The patient opted for surgical treatment. During surgery, a portion of the skull that was assumed to be on the lesion was exposed via a linear skin incision. Neither substantial changes nor deficits were found on the skull surface, but a part of the surface that was located around the lateral end of the lesion contained a reddish-blue tinge, suggesting the thinning of the outer table of the skull. The bluish part of the outer table of the skull was removed to expose a membrane of a sac-like structure which was filled with liquid. The membrane was not pulsating. The sac had several communications with the diploic veins that we assumed to be feeding vessels. By interrupting these inflows, the sac deflated. A section at the end of the sac was cut out and sent for histological examination. There was copious bleeding from multiple interdiploic venous channels, and it was controlled by the bone wax. On the other hand, bleeding from the edge of the sac was not so heavy, suggesting that main feedings no longer remained. The edge was then ligated and the scalp was closed in layers.

**Fig. 5:**
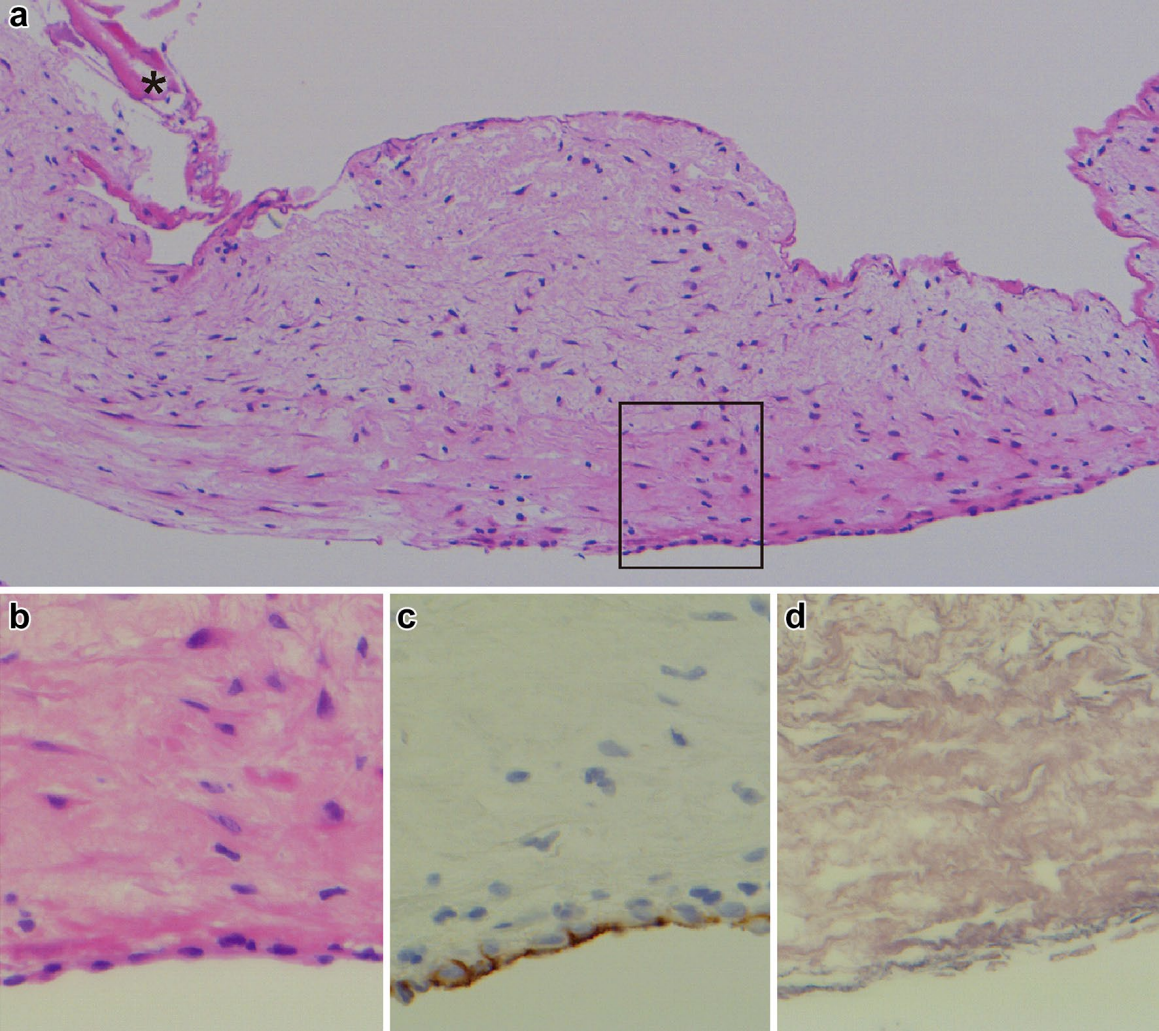
Histological images of a membranous wall of varix-like venous sac. The upper image was in a low power field (**a** Hematoxylin and eosin staining, ×150), and the lower images were in high power fields (**b** Hematoxylin and eosin staining; **c** CD31 staining; **d** silver staining, ×400). The membrane mainly consisted of a thick layer of fibrous tissues. Osseous tissues (*asterisk*) were attached to the upper side of the membrane (**a**). The other side was lined by a single layer of endothelial cells (**b**), which showed positive CD31 staining (**c**). Silver staining of the membrane was negative, indicating the absence of elastic laminae (**d**)

Histopathological study of the lesion showed a membranous wall that consisted mainly of fibrous tissues with scattered spindle cells (Fig. 5a). No oncocytes were present. The membranous wall was considered to be a dilated venous wall as it contained a single layer of luminal endothelial cells (Fig. 5b) that was positive for CD31 staining (Fig. 5c), but lacked elastic laminae after silver staining (Fig. 5d).

The post-operative course of the patient was uneventful. MRI showed that there was an intensity change in the inside of the sac on T2-weighted images. The main part of the inside of the sac showed low intensity prior to surgery (Fig. 3d) but changed to high intensity after surgery (Fig. 3e), suggesting that it had been filled with thrombi. In addition, magnetic resonance venography showed that there was no longer any blood inflow into the sac (Fig. 4c, d). At a follow-up time-point of 15 months, there was no recurrence of abnormal venous flow.

## Discussion

The diploic venous system is connected with both the intra- and extracranial venous systems via the dural sinuses and the emissary veins, respectively. It is involved in various vascular anomalies. Although its occurrence is rare, abnormal arteriovenous shunts to the diploic vein can be lethal. For instance, dural arteriovenous fistulas can cause intracerebral hemorrhage (Yako et al. 2016) and subdural hematoma (Rivera-Lara et al. 2015). Scalp arteriovenous malformation, which causes scalp swelling, headache, and recurrent bleeding (Chowdhury et al. 2013), can also drain to the diploic vein (Bekelis et al. 2011). On the other hand, the most common diploic venous anomaly is sinus pericranii. It is considered as an abnormal communication between the intra- and extracranial venous systems (Sakai et al. 1997) and typically presents as a soft scalp swelling. In some cases, this is accompanied by local pain, headache, nausea, and vertigo (Akram et al. 2012). In regards to other venous anomalies, there have been reports of cases of subepicranial varix with connections to diploic veins, but these varices had no connections to intracranial venous sinuses (Asano et al. 2000; Mori et al. 1976).

The patient in this case report also had a varix-like venous sac in the diploe that was continuous with the diploic veins, but it was covered by the outer table of the skull and isolated from the extracranial structures. Because of this distinct lack of communications with the extracranial venous system, it belonged to neither sinus pericranii nor subepicranial varix. Accordingly, it was diagnosed as another entity of venous anomaly that should be termed “intradiploic varix”. This entity has not reported previously maybe because it should be asymptomatic in general.

On the other hand, from a pathological viewpoint, as our histological study showed the endothelial lining, which is typically observed in sinus pericranii (Bollar et al. 1992), their etiological backgrounds may be similar. Although the etiology of sinus pericranii is still unknown, two possible etiologies have been postulated: congenital and traumatic. As sinus pericranii is frequently associated with intracranial developmental venous anomalies or other anomalies, it suggests a congenital cause, such as transient venous hypertension during the late embryonic period (Nomura et al. 2006). On the other hand, some patients developed sinus pericranii without congenital anomalies after head trauma. In this case, it suggests that sinus pericranii has an acquired pathophysiology (David et al. 1998), but the causal trauma is frequently too slight to be noticed (Bollar et al. 1992). The patient in our case did not have any other venous anomalies but may have suffered minor trauma in the past.

Another significant point in our case was the expansion of the lesion during the 5-year period and the surrounding hyperosteosis. Assuming that the pathology is similar to that of sinus pericranii, the intravarix pressure would increase and decrease intermittently in response to the intracranial pressure. For sinus pericranii, this intermittent pressure change leads to a change in the size of the scalp swelling. However, in our case, because the venous sac was covered by the cortical bone, it may have gradually eroded the diploe and simultaneously induced reactive hyperosteosis.

In regards to the treatment, the patient opted for surgery despite the lack of symptoms because the lesion had been expanding and it was necessary to differentiate it from neoplastic diseases. Intradiploic varices that have a low risk of being lethal do not always need to be treated, but in cases of expanding lesions such as in our patient, treatment is recommended to avoid further erosion of the skull. Aside from direct surgical interventions such as resection or venous occlusion, endovascular occlusion may also be an option, based on the history of sinus pericranii treatment. However, there is still insufficient evidence to support it (Brook et al. 2009).

## Conclusions

The abnormal connection between the intracranial and the diploic venous systems via the large venous sac was diagnosed as a new entity of venous anomaly that should be termed “intradiploic varix”. It could be considered pathologically similar to sinus pericranii. This case suggests that invasive treatments are recommended for expanding lesions, even if they are asymptomatic.

## Abbreviations

CT: computed tomography
MRI: magnetic resonance imaging
